# Global skin colour prediction from DNA

**DOI:** 10.1007/s00439-017-1808-5

**Published:** 2017-05-12

**Authors:** Susan Walsh, Lakshmi Chaitanya, Krystal Breslin, Charanya Muralidharan, Agnieszka Bronikowska, Ewelina Pospiech, Julia Koller, Leda Kovatsi, Andreas Wollstein, Wojciech Branicki, Fan Liu, Manfred Kayser

**Affiliations:** 1 Department of Biology, Indiana University Purdue University Indianapolis (IUPUI), Indianapolis, IN USA; 2 Department of Genetic Identification, Erasmus MC University Medical Centre Rotterdam, Rotterdam, The Netherlands; 3 Department of Dermatology, Collegium Medicum of the Jagiellonian University, Kraków, Poland; 4 Faculty of Biology and Earth Sciences, Institute of Zoology, Jagiellonian University, Kraków, Poland; 5 Malopolska Centre of Biotechnology, Jagiellonian University, Kraków, Poland; 6 Laboratory of Forensic Medicine and Toxicology, School of Medicine, Aristotle University of Thessaloniki, Thessaloniki, Greece; 7 Section of Evolutionary Biology, Department of Biology II, University of Munich LMU, Planegg-Martinsried, Germany; 8 Central Forensic Laboratory of the Police, Warsaw, Poland; 9 Key Laboratory of Genomic and Precision Medicine, Beijing Institute of Genomics, Chinese Academy of Sciences, Beijing, China; 10 University of Chinese Academy of Sciences, Beijing, China

## Abstract

Human skin colour is highly heritable and externally visible with relevance in medical, forensic, and anthropological genetics. Although eye and hair colour can already be predicted with high accuracies from small sets of carefully selected DNA markers, knowledge about the genetic predictability of skin colour is limited. Here, we investigate the skin colour predictive value of 77 single-nucleotide polymorphisms (SNPs) from 37 genetic loci previously associated with human pigmentation using 2025 individuals from 31 global populations. We identified a minimal set of 36 highly informative skin colour predictive SNPs and developed a statistical prediction model capable of skin colour prediction on a global scale. Average cross-validated prediction accuracies expressed as area under the receiver-operating characteristic curve (AUC) ± standard deviation were 0.97 ± 0.02 for Light, 0.83 ± 0.11 for Dark, and 0.96 ± 0.03 for Dark-Black. When using a 5-category, this resulted in 0.74 ± 0.05 for Very Pale, 0.72 ± 0.03 for Pale, 0.73 ± 0.03 for Intermediate, 0.87±0.1 for Dark, and 0.97 ± 0.03 for Dark-Black. A comparative analysis in 194 independent samples from 17 populations demonstrated that our model outperformed a previously proposed 10-SNP-classifier approach with AUCs rising from 0.79 to 0.82 for White, comparable at the intermediate level of 0.63 and 0.62, respectively, and a large increase from 0.64 to 0.92 for Black. Overall, this study demonstrates that the chosen DNA markers and prediction model, particularly the 5-category level; allow skin colour predictions within and between continental regions for the first time, which will serve as a valuable resource for future applications in forensic and anthropologic genetics.

**Electronic supplementary material:**

The online version of this article (doi:10.1007/s00439-017-1808-5) contains supplementary material, which is available to authorized users.

## Introduction

Predicting phenotypes from genotypes is a component of complex genetics that has etched its way into many disciplines including personalized medicine, forensic genetics, anthropological genetics, and consumer genetics, depending on the particular phenotype that is predicted from DNA information. The ability to predict human phenotypes with genetic markers has been of continual interest and significant progress has been made, not only in these applied disciplines, but also to more fundamental genetics researchers as it paves the way to find out why certain DNA markers are found to be associated with certain phenotypic traits.

In the case of eye colour, one of the first physical appearance traits to be studied for predictability from DNA, elucidation of its associated DNA markers (Duffy et al. 2007; Eiberg et al. 2008; Frudakis et al. 2003, 2007; Graf et al. 2005; Han et al. 2008; Kanetsky et al. 2002; Kayser et al. 2008; Liu et al. 2010; Posthuma et al. 2006; Rebbeck et al. 2002; Sturm et al. 2008; Sulem et al. 2007, 2008; Zhu et al. 2004), and subsequent step-wise ranking on how suitable they were for phenotype prediction (Liu et al. 2009) led to the introduction, further development, and forensic validation of the IrisPlex system (Chaitanya et al. 2014; Walsh et al. 2011a, b, 2012). It achieved average prediction accuracies, expressed as Area Under the receiver-operating characteristic Curve (AUC), of 0.94 for blue, 0.95 brown, and 0.74 for intermediate (Walsh et al. 2014), and was used in practical applications (Dembinski and Picard 2014; Kastelic et al. 2013; Yun et al. 2014). Moreover, it was demonstrated that for the SNP with the highest prediction rank, rs12913832 from intron 86 of the *HERC2* gene, the two alleles act as a molecular switch regulating expression of the nearby *OCA2* gene via long-distance enhancer function (Visser et al. 2012).

For human hair colour, gene mapping studies also identified numerous highly associated SNPs (Box et al. 1997; Branicki et al. 2007, 2008a; Fernandez et al. 2008; Flanagan et al. 2000; Graf et al. 2005; Grimes et al. 2001; Han et al. 2008; Harding et al. 2000, 2002, Kanetsky et al. 2004; Mengel-From et al. 2009; Pastorino et al. 2004; Rana et al. 1999; Sulem et al. 2007, 2008; Valenzuela et al. 2010; Valverde et al. 1995; Voisey et al. 2006), 22 of which proved decidedly predictive for hair colour categories (Branicki et al. 2011). From this, and previous eye colour knowledge, the HIrisPlex system was developed and forensically validated for combined eye and hair colour prediction from DNA achieving AUCs of 0.92 for red, 0.85 for black, 0.81 for blond, and 0.75 for brown (Draus-Barini et al. 2013; Walsh et al. 2013, 2014). The HIrisPlex DNA markers and prediction models were used in what has been referred to as the oldest forensic case to date—King Richard III (King et al. 2014) as well as in anthropological estimations of ancestral physical appearance (Cassidy et al. 2016; Gallego-Llorente et al. 2016; Gamba et al. 2014; Jones et al. 2015; Martiniano et al. 2016; Olalde et al. 2015).

Skin coloration, however, is a more difficult physical appearance trait to examine genetically and to elucidate how its associated markers can be ranked for prediction, due to its population specific influence (Jablonski and Chaplin 2000, 2013). The maximal skin colour difference between people from different continents, as a result of environmental adaptation and consequence of the out of Africa migration (Liu et al. 2006), leads to a restriction in gene mapping studies. Genome-wide association studies (GWASs) are typically conducted in genetically homogeneous samples to avoid, as much as possible, the false positives that may be produced due to different genetic background between study samples. Therefore, GWASs on skin colour that are performed within continental groups such as Europeans (Han et al. 2008; Liu et al. 2015; Sulem et al. 2008) or South Asians (Edwards et al. 2010; Stokowski et al. 2007) basically identified a list of SNPs explaining subtle skin colour variation within each continental group, but in principle cannot reveal a complete list of skin colour-associated SNPs. Consequently, a previously described prediction model built on exclusively European subjects using SNPs identified in a European skin colour GWAS (Liu et al. 2015) had no power to predict skin colour differences between non-European continents, such as East Asia, Africa, and Native Americans, where considerable skin colour differences exist (Liu et al. 2015). Conversely, previously described skin colour prediction models developed from multi-ethnic data (Maroñas et al. 2014; Valenzuela et al. 2010) had no power to predict skin colour differences within continental groups, such as within Europeans. Noteworthy, a model combining many of these associated SNPs, allowing both DNA-based skin colour prediction within and between continents, has not been described thus far.

The early attempts at predicting skin colour phenotypes from DNA were highly limited in their outcomes (Mushailov et al. 2015; Spichenok et al. 2011; Valenzuela et al. 2010). More recently, Maroñas et al. (2014) published a skin colour prediction study examining 59 pigmentation-associated SNPs in two populations, Africans and Europeans as well as a subset of admixed African-Europeans. Upon training their Bayesian classifier model with a set of 280 individuals, the authors decided on a set of 10 SNPs that together achieved AUC values of 0.999 for white, 0.966 for black, and 0.803 for intermediate skin colour. However, due to the low numbers used in the validation set (*n* = 118) and the limited populations and individuals studied, it is worthwhile to re-examine these prediction accuracies on a more extensive global scale. Moreover, the previous studies treated Europeans as one group in their prediction analysis (i.e., light skin colour), thereby ignoring the level of skin colour variation from very pale via pale to intermediate that exists among people of European descent.

In an effort to circumvent the current limitations in predicting skin colour from DNA, we tested a large number of SNPs previously associated with human pigmentation traits in a considerable number of individuals from worldwide populations to investigate their skin colour predictive value. As skin colour phenotypes, we used skin types obtained from the Fitzpatrick scale, which is of widespread use in dermatology research and clinical practice. The Fitzpatrick scale groups individuals based on both visually perceived skin colour and skin sensitivity to sun, including tanning ability; the latter being important to differentiate between Europeans of differing light skin tones. We selected a set of the most skin colour informative SNP predictors and built a statistical model for predicting skin colour from DNA on a global scale using 3 and 5 skin colour categories. In addition, we directly compared the prediction outcomes of our newly developed skin colour model with a previously developed model using a separate set of global individuals not previously involved in SNP predictor selection, model building, and model testing.

## Materials and methods

### Samples and skin colour phenotyping

We used 1159 individuals from Southern Poland, 347 individuals from Ireland, 119 from Greece, and 329 individuals living in the USA (parental place of birth for many of these individuals is outside the US; these include Nigeria, Mexico, Argentina, Columbia, India, Bangladesh, Cuba, Palestine, Canada, China, Honduras, Germany, Philippines, Russia, Sudan, Japan, Saudi Arabia, Pakistan, El Salvador, Spain, Haiti, South Korea, Vietnam—see online resource information 1). Informed consent was obtained from all individual participants included in the study and was approved by ethical committees of the cooperating institutions. Also included in this study were 71 individuals from the HGDP-CEPH (Rosenberg 2006) set, i.e., from Senegal (*n* = 21), Nigeria (*n* = 21), Kenya (*n* = 11), and Papua New Guinea (*n* = 17). In total, 2025 individuals were genotyped.

In terms of phenotyping, skin colour classifications followed the Fitzpatrick scale (Fitzpatrick 1988). The scale represents a dermatological assessment to estimate the response of different types of skin to UV light; therefore, it takes into account visual perception of skin colour, as well as tanning ability (Fitzpatrick 1988). It is commonly used by medical practitioners for the classification of a persons skin type, ranging from skin type 1 (pale white skin—no tanning ability), 2 (white skin—minimal tanning ability), 3 (light brown skin—tanning ability), 4 (moderate brown skin—tanning ability), and 5 (dark brown skin—tanning ability) to skin type 6 (deeply pigmented dark brown to black skin)—see online resource information 2. The Polish samples were assessed for their Fitzpatrick skin type by an experienced dermatologist (AB) at sample collection. The Irish, Greek, and US individuals were also assessed by the same dermatologist upon consultation of photographic imagery, and a detailed questionnaire on their ability to tan. Images were taken approximately 20 cm from the forearm of the individual using a Nikon D5300 and R1 ring flash with the following settings: Focus 22, Aperture 1/125, ISO 200. Therefore, all individuals collected were assigned an objective Fitzpatrick scale designation by the same qualified dermatologist avoiding the subjective designations that the volunteers themselves would provide in questionnaire data. For the HGDP-CEPH samples, for which no individual skin colour phenotype information was available, Fitzpatrick scales 6 was assigned as assumed from population knowledge of these African and New Guinean groups, as people living in these geographic regions only have very dark-black skin colour. The 6 Fitzpatrick scales were then re-classified into 5 final skin colour prediction categories for further analyses, i.e., Very Pale (6% of all samples used), Pale (44%), Intermediate (42%), Dark (3%), and Black (5%) by condensing the Fitzpatrick categories 3 and 4 into the Intermediate prediction category and leaving all other categories the same. Categories 3 and 4 of the Fitzpatrick scale are considered very close dermatologically; therefore, it was deemed acceptable to combine these categories for the prediction training of this skin colour model. In a 3-category scale, we grouped Fitzpatrick scale 1–4 Into Light (92%), scale 5 Into Dark (3%), and scale 6 into Dark-Black (5%). Henceforth, the term skin colour category with reference to the categories predicted shall be used for reasons of simplicity in the text; however, it does include not only the visual perception of skin colour but also the ability or lack of to tan. Further information on the Fitzpatrick scale can be found in online resource information 2.

For directly comparing our findings with those from Maroñas et al. (2014), individuals from an independent sample set (*n* = 194, 17 different populations from Europe, Middle-East, Africa, and Asia) not used in the previous marker ascertainment, model building, or testing, were predicted for skin colour using both models, the one established here, and the one proposed by Maroñas et al. (2014). For this, the same skin colour phenotyping approach as described by Maroñas et al. (2014) was used to make the study outcomes directly comparable. L*ab groups were designated a simple 3-category definition of White, Intermediate, and Black based on groups of L*ab values. The spectrometer values were: L*ab = 74.14–60.36 for White, comprising 132 samples; 59.32–40.04 for Intermediate, comprising 43 samples; 39.75–29.99 for Black, comprising 20 samples.

### SNP assessment, genotyping, & statistical analyses

This study examined 2025 individuals for 77 single-nucleotide polymorphisms (SNPs) from 37 genetic loci that were associated with human pigmentation variation, skin colour in particular, in the previous studies (see Table 1 for more details). SNPs were genotyped using SNaPshot (Life Technologies) multiplexes designed and optimized very similar to those described elsewhere (Walsh et al. 2011b, 2013). A subset of 53 SNPs (see Table 1) from 24 genes were selected for further assessment based on their independent contribution (*R* ^*2*^ *p* value <0.05 uncorrected) towards categorical skin colour prediction, while factoring in sex and population. Finally, the Akaike Information Criterion (AIC) was used for determining optimal SNP selection from the 53 SNPs, which resulted in 36 SNPs from 16 genes (*SLC24A5* rs1426654, *IRF4* rs12203592, *MC1R* rs1805007, rs1805008, rs11547464, rs885479, rs228479, rs1805006, rs1110400, rs1126809, rs3212355, *OCA2* rs1800414, rs1800407, rs12441727, rs1470608, rs1545397 *SLC45A2* rs16891982, rs28777, *HERC2* rs1667394, rs2238289, rs1129038, rs12913832, rs6497292, *TYR* rs1042602, rs1393350, *RALY* rs6059655, *DEF8* rs8051733, *PIGU* rs2378249, *ASIP* rs6119471, *SLC24A4* rs2402130, rs17128291, rs12896399, *TYRP1* rs683, *KITLG* rs12821256, *ANKRD11* rs3114908, and *BNC2* rs10756819).

**Table 1:** DNA variant information for 77 SNPs previously associated with human pigmentation variation including their location, citations, as well as skin colour association and prediction ranking details obtained from the present study

	SNP	Chromosome	Gene	Alleles	BP (GRCh38)	Reference pigmentation association	Skin colour correlation [*r* ^2^ (*p* value)]*	Ranking in final model	Coefficients (fitted glm)	*P* value
1	rs6679651	1	HIST2H2BF	C/T	149,757,453		ns			
2	rs12233134	2	EFR3B	C/T	25,106,146	Quillen et al. (2012)	ns			
3	rs40132	5	SLC45A2	A/G	33,950,597	Nan et al. (2009)	ns			
4	rs16891982	5	SLC45A2	C/G	33,951,587	Liu et al. (2009); Stokowski et al. (2007); Valenzuela et al. (2010); Branicki et al. (2011)	0.142 (8.13e-58)	5	0.27912209	1.72E-08
5	rs2287949	5	SLC45A2	C/T	33,954,405	Stokowski et al. (2007)	0.006 (0.004)			
6	rs28777	5	SLC45A2	G/T	33,958,853	Branicki et al. (2011); Duffy et al. (2010); Han et al. (2008)	0.097 (3.14E-40)	24	8.65E-02	7.57E-02
7	rs26722	5	SLC45A2	A/G	33,963,764	Han et al. (2008); Liu et al. (2009); Stokowski et al. (2007)	ns			
8	rs6867641	5	SLC45A2	C/T	33,985,751	Graf et al. (2007)	ns			
9	rs13289	5	SLC45A2	C/G	33,986,303	Graf et al. (2007); Han et al. (2008); Maroñas et al. (2014)	0.0114 (5.8E-05)			
10	rs1936208	6	Intergenic between ATP5F1P6 and LOC100129554	C/T	139,644,247		ns			
11	rs12203592	6	IRF4	C/T	396,320	Branicki et al. (2011); Han et al. (2008); Liu et al. (2009); Praetorius et al. (2013)	0.0201 (5.18e-09)	2	−0.17565966	1.97E-12
12	rs4959270	6	LOC105374875	A/C	457,747	Branicki et al. (2011); Han et al. (2008); Sulem et al. (2007)	ns			
13	rs477823	7	<NA>	G/T	63,287,722		0.0068 (0.001)			
14	rs1385229	8	C8orf37-AS1	A/G	95,759,318		ns			
15	rs10756819	9	BNC2	A/G	16,858,085	Liu et al. (2015); Visser et al. (2014)	0.021 (2.48E-09)	36	1.32E-03	9.46E-01
16	rs683	9	TYRP1	A/C	12,709,304	Branicki et al. (2011); Liu et al. (2009)	0.0096 (4.6E-05)	32	1.70E-02	3.83E-01
17	rs376397	10	GATA3	A/G	8,061,334		ns			
18	rs10443915	10	PRKG1	A/T	52,060,818		ns			
19	rs12765852	10	PRKG1	C/T	52,061,566		ns			
20	rs10831496	11	GRM5	A/G	88,824,822	Nan et al. (2009)	ns			
21	rs4936890	11	Intergenic between OR10G7 and OR10D5P	A/G	124,044,034		0.0113 (1.5E-05)			
22	rs35264875	11	TPCN2	A/T	69,078,930	Jacobs et al. (2015); Sulem et al. (2008); Valenzuela et al. (2010); Zhang et al. (2013)	0.0034 (0.016)			
23	rs1042602	11	TYR	A/C	89,178,527	Branicki et al. (2011); Jonnalagadda et al. (2016); Sulem et al. (2007)	0.0025 (0.04)	12	−0.06223707	3.52E-03
24	rs1393350	11	TYR	A/G	89,277,877	Han et al. (2008); Liu et al. (2009); Nan et al. (2009); Sulem et al. (2007)	0.0109 (1.8E-05)	21	−5.60E-02	5.96E-02
25	rs1126809	11	TYR	A/G	89,284,793	Branicki et al. (2011); Duffy et al. (2010); Sulem et al. (2007)	0.015 (2.2E-06)	19	−0.08357710	2.28E-02
26	rs642742	12	KITLG	A/G	88,905,968	Jonnalagadda et al. (2016)	0.0533 (5.2E-21)			
27	rs12821256	12	KITLG	C/T	88,934,557	Branicki et al. (2011); Guenther et al. (2014); Sulem et al. (2007)	0.0024 (0.046)	33	−1.52E-02	6.53E-01
28	rs3782974	13	DCT	A/T	94,440,641	Lao et al. (2007)	0.0095 (6.6E-05)			
29	rs2050537	13	HS6ST3	C/T	96,608,646		ns			
30	rs4983161	14	<NA>	A/T	19,726,716		0.007 (0.001)			
31	rs12896399	14	LOC105370627 (upstream of SLC24A4)	G/T	92,307,318	Han et al. (2008); Liu et al. (2009); Sulem et al. (2007)	0.011 (1.8E-05)	29	-2.55E-02	2.08E-01
32	rs2402130	14	SLC24A4	A/G	92,334,858	Branicki et al. (2011); Sulem et al. (2007)	0.027 (6.8E-12)	27	3.98E-02	1.09E-01
33	rs17128291	14	SLC24A4	A/G	92,416,481	Liu et al. (2015)	0.0147 (7.28E-07)	28	−3.91E-02	1.30E-01
34	rs12914268	15	<NA>	A/G	22,150,292		ns			
35	rs1129038	15	HERC2	A/G	28,111,712	Liu et al. (2010); Mengel-From et al. (2010)	0.092 (1.77E-37)	17	0.10536412	8.38E-03
36	rs12913832	15	HERC2	A/G	28,120,471	Branicki et al. (2011); Duffy et al. (2007); Kayser et al. (2008); Liu et al. (2009); Mengel-From et al. (2010); Sturm et al. (2008); Sulem et al. (2007); Visser et al. (2012)	0.091 (9.9E-37)	20	8.12E-02	3.45E-02
37	rs2238289	15	HERC2	C/T	28,208,068	Mengel-From et al. (2009), (2010)	0.033 (5.24E-14)	15	−0.11378297	8.00E-03
38	rs8182028	15	HERC2	C/T	28,222,788	Liu et al. (2009)	ns			
39	rs3940272	15	HERC2	A/C	28,223,576	Eiberg et al. (2008)	ns			
40	rs6497292	15	HERC2	A/G	28,251,048	Kayser et al. (2008); Liu et al. (2009)	0.075 (2.29E-30)	30	5.79E-02	2.27E-01
41	rs16950941	15	HERC2	A/G	28,257,597	Liu et al. (2009)	ns			
42	rs1667394	15	HERC2	A/G	28,285,035	Duffy et al. (2007); Kayser et al. (2008); Liu et al. (2009); Mengel-From et al. (2010); Sturm et al. (2008); Sulem et al. (2007)	0.052 (1.15E-21)	6	0.16017374	4.70E-08
43	rs1473917	15	LOC101927079	C/T	22,067,210		ns			
44	rs1545397	15	OCA2	A/T	27,942,625	Edwards et al. (2010)	0.0166 (2.27E-07)	34	−1.03E-02	7.51E-01
45	rs1800414	15	OCA2	A/G	27,951,890	Donnelly et al. (2012); Edwards et al. (2010)	0.047 (2.79E-19)	4	−0.53990294	6.12E-11
46	rs1800407	15	OCA2	A/G	27,985,171	Branicki et al. (2011); Donnelly et al. (2012); Duffy et al. (2010); Liu et al. (2009)	0.007 (4.4E-04)	8	−0.19827349	1.20E-06
47	rs1800401	15	OCA2	C/T	28,014,906	Branicki et al. (2008b); Duffy et al. (2007)	0.0054 (0.005)			
48	rs12441727	15	OCA2	A/G	28,026,628	Liu et al. (2009)	0.0047 (0.005)	25	6.03E-02	8.23E-02
49	rs1448485	15	OCA2	A/C	28,037,594	Duffy et al. (2007); Kayser et al. (2008); Liu et al. (2009)	ns			
50	rs16950821	15	OCA2	A/G	28,038,360	Branicki et al. (2011)	0.037 (3.6E-15)			
51	rs1470608	15	OCA2	A/C	28,042,974	Branicki et al. (2011); Mengel-From et al. (2009)	0.063 (1.04E-25)	31	−3.79E-02	2.66E-01
52	rs7495174	15	OCA2	A/G	28,099,091	Branicki et al. (2009); Donnelly et al. (2012); Duffy et al. (2007); Edwards et al. (2010); Liu et al. (2009)	ns			
53	rs1426654	15	SLC24A5	A/G	48,134,286	Lamason et al. (2005); Stokowski et al. (2007); Sturm and Larsson (2009); Valenzuela et al. (2010)	0.15 (1.19E-59)	1	0.52412661	1.92E-23
54	rs11076649	16	AFG3L1P	C/G	89,992,927		0.0058 (0.002)			
55	rs3114908	16	ANKRD11	A/G	89,317,316	Law et al. (2015)	0.0201 (9.8E-09)	35	3.93E-03	8.56E-01
56	rs8049897	16	DEF8	A/G	89,957,793	Han et al. (2008); Jin et al. (2012)	0.022 (1.5E-09)			
57	rs8051733	16	DEF8	A/G	89,957,797	Law et al. (2015)	0.029 (2.7E-12)	16	−0.06364481	8.16E-03
58	rs164741	16	DPEP1	C/T	89,625,889	Han et al. (2008); Nan et al. (2009)	0.015 (2.76E-07)			
59	rs2239359	16	FANCA	C/T	89,783,071		ns			
60	rs3212355	16	MC1R	C/T	89,917,969	Valenzuela et al. (2010)	0.0206 (2.89E-08)	22	2.00E-01	6.14E-02
61	rs312262906 (N29insA)	16	MC1R	INDEL -/insA	89,919,341	Branicki et al. (2011)	0.0085 (1.2E-04)			
62	rs1805005	16	MC1R	G/T	89,919,435	Branicki et al. (2011); Duffy et al. (2010); Stokowski et al. (2007); Sturm et al. (2003)	ns			
63	rs1805006	16	MC1R	A/C	89,919,509	Branicki et al. (2011); Duffy et al. (2010); Liu et al. (2015)	0.003 (2.2E-02)	13	−0.31065309	5.63E-03
64	rs2228479	16	MC1R	A/G	89,919,531	Branicki et al. (2011); Sturm et al. (2003)	0.019 (7.45E-09)	11	−0.10915180	1.70E-03
65	rs11547464	16	MC1R	A/G	89,919,682	Branicki et al. (2011); Duffy et al. (2010)	0.0071 (4.6E-04)	9	−2.96E-01	5.06E-04
66	rs1805007	16	MC1R	C/T	89,919,708	Branicki et al. (2011); Duffy et al. (2010); Sulem et al. (2007)	0.0268 (1.28E-11)	3	−0.28231475	5.92E-12
67	rs201326893 (Y152OCH)	16	MC1R	C/A	89,919,713	Branicki et al. (2011)	ns			
68	rs1110400	16	MC1R	C/T	89,919,721	Branicki et al. (2011)	0.0037 (1.1E-02)	18	−0.20059956	1.02E-02
69	rs1805008	16	MC1R	C/T	89,919,735	Branicki et al. (2011); Sulem et al. (2007)	0.021 (9.2E-10)	7	−0.19994906	1.25E-07
70	rs885479	16	MC1R	A/G	89,919,746	Branicki et al. (2011); Sturm et al. (2003)	0.0326 (7.63E-14)	10	−0.16300889	5.42E-04
71	rs1805009	16	TUBB3	C/G	89,920,137	Branicki et al. (2011); Duffy et al. (2010)	ns			
72	rs333113	17	SPNS2	C/G	4,497,060		0.013 (2.41E-06)			
73	rs6119471	20	ASIP	C/G	34,197,405	Hart et al. (2013)	0.214 (4.76E-85)	26	9.27E-02	9.51E-02
74	rs2424984	20	ASIP	C/T	34,262,568	Valenzuela et al. (2010)	0.044 (2.06E-17)			
75	rs1885120	20	MYH7B	C/G	34,989,185	Liu et al. (2015)	0.003 (0.039)			
76	rs2378249	20	PIGU	A/G	34,630,285	Branicki et al. (2011)	0.008 (1.4E-04)	23	−4.76E-02	7.36E-02
77	rs6059655	20	RALY	A/G	34,077,941	Jacobs et al. (2015); Liu et al. (2015)	0.008 (4.2E-04)	14	−0.11371271	7.23E-03

*ns* not significant

After quality control due to some missing genotypes for the full 36 SNP set, Multinomial Logistic Regression (MLR) modelling was performed for the prediction of categorical skin colour based upon a set of 1423 individuals. Details of the model for the prediction analysis follow studies on eye (Liu et al. 2009; Walsh et al. 2011b) and hair (Branicki et al. 2011; Walsh et al. 2013) colour prediction previously performed. In brief, categorical skin colour, based on five categories (and also three categories), is designated *y*, and is determined by genotype × (number of minor alleles per *k*) of *k* SNPs. For the 5-category designation, π1, π2, π3, π4, and π5 denote the probability of Very Pale, Pale, Intermediate, Dark, and Dark-Black, respectively. To investigate the performance of the 36 skin colour-associated SNPs in a prediction model overall, cross validations were conducted in 1000 randomized replicates; in each replicate, 80% individuals were used as the new training set (*n* = 1138) and the remaining samples were used as the testing set (*n* = 285). AUC values were derived from the testing set, and the average AUC values and the standard deviation were reported. AUC values of 0.5 designate a random prediction, whereas values closer to 1 indicate perfect prediction accuracy. Prediction results were produced for five categories as previously named and for three categories; Light (collapsing Very Pale, Pale, and Intermediate), Dark and Dark-Black to illustrate a 3-category grouping. For this study, skin colour prediction probabilities were generated for the test set with the highest probability leading to the most probable prediction for skin colour for each individual.

For comparing our findings with those of Maroñas et al. (2014), an independent set of individuals (*n* = 194) described as the ‘model comparison set’ were genotyped for the 36 skin colour SNP predictors identified in this study as well as the 10 skin colour SNP predictors proposed by Maroñas et al. (2014) study, allowing a direct comparison of the prediction performance of these two models and their own sets of DNA predictors. For this, the 10 SNPs proposed by Maroñas et al. (2014); *KITLG* rs10777129, *SLC45A2* rs13289 and rs16891982, *TYRP1* rs1408799, *SLC24A5* rs1426654, *OCA2* rs1448484, *SLC24A4* rs2402130, *TPCN2* rs3829241, *ASIP* rs6058017, and rs6119471 were genotyped in these 194 samples using SNaPshot (Life Technologies) multiplexing. The Naïve Bayes skin classifier (http://mathgene.usc.es/snipper/skinclassifier.html) was used to predict each individual using the websites requested genotype input. An assessment of the models performance for categorical skin colour prediction was made on the full set of 194 individuals using a confusion matrix of prediction versus observed phenotype, which yielded AUC, Sensitivity, Specificity, Positive Predictive Value (PPV), and Negative Predictive Value of the model. To directly compare to the performance of the 36 markers proposed by this group, the same individuals were assessed using this study’s proposed 3-category model using the same phenotype scale as recommended by Maroñas et al. (2014). Therefore, the only differing factor was the performance of the Maroñas et al. (2014) skin colour classifier and the 36-marker model proposed in this study for the prediction of categorical skin colour.

All statistical analyses were performed with the R statistics software (R Core Team 2013), using packages MASS (Venables 2002), mlogit (Croissant 2013), ROCR (Sing et al. 2005), pROC (Robin et al. 2011), and caret (Kuhn et al. 2016).

## Results and discussion

### Selection of skin colour SNP predictors

We tested 77 previously pigmentation-associated SNPs from 37 genetic loci (see Table 1 for more information) in 2025 individuals for their value in predicting skin colour from DNA using the Fitzpatrick scale as a phenotype classification system. A partial correlation correcting for sex and population ancestry yielded a subset of 53 SNPs that were statistically significantly associated with the categorical skin colour scale in these individuals (*p* < 0.05 uncorrected) (see Table 1 for associated SNPs).

Next, model selection was performed on the resulting 53 SNPs using the Akaike Information Criterion (AIC) to estimate the information lost using certain combinations of SNPs, resulting in a balance between goodness of fit for the prediction model and number of SNP inclusions. This approach led to a final set of 36 SNPs from 16 genes (see “Materials and methods”) that were selected for final prediction modelling. Only individuals with a complete list of genotypes for the 36 SNPs could be used for prediction modelling; this led to a decrease in final numbers from 2025 to 1423 individuals.

### Prediction modelling of skin colour phenotypes from genotypes

**Fig. 1:**
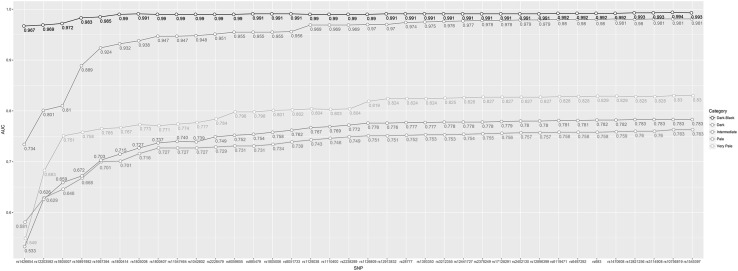
Illustration of the accumulative contribution of each of the selected 36 SNP predictors towards AUC prediction accuracy of 5 skin colour categories based on the full set of 1423 individual. SNP predictors were added to the prediction model one by one in the sequential order from highest to lowest prediction rank.* Each colour-coded line* represents one of the 5 DNA-predicted* skin colour* categories.* Skin colour* phenotyping was via skin types derived from the Fitzpatrick scale

**Fig. 2:**
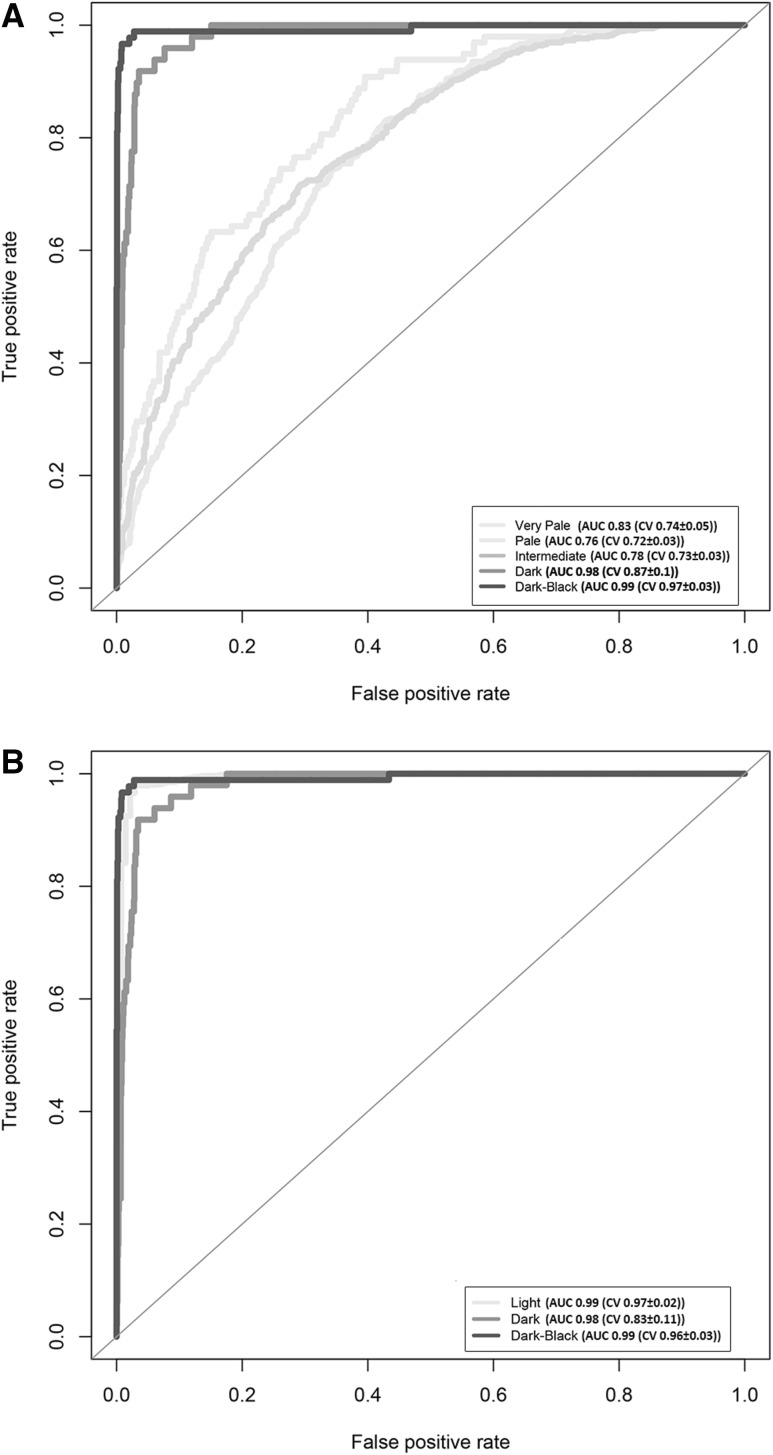
Illustration of the prediction performance of the set of 36 SNPs for the 5-category (**a**) and the 3-category (**b**) skin colour prediction model using ROC curves with AUC estimates (including the cross-validated measures) using the full training set of 1423 individuals from 29 populations.* Skin colour* phenotyping was via skin types derived from the Fitzpatrick scale

MLR modelling was performed on this 36-SNP set in 1423 individuals using the following categories: Very Pale *n* = 98, Pale *n* = 631, Intermediate *n* = 555, Dark *n* = 49, and Dark-Black *n* = 90. To illustrate the breakdown of each SNP’s contribution towards categorical skin colour prediction using 100% of the individuals (*n* = 1423), each SNP is added sequentially and their collated prediction effect in terms of AUC is estimated, as shown in Fig. 1. To describe the final model chosen, the α and β for each SNP were derived from the full set of 1423 individuals (Male *n* = 556, Female *n* = 867; Very Pale *n* = 98, Pale *n* = 631, Intermediate *n* = 555, Dark *n* = 49, and Dark-Black *n* = 90) for each skin colour category, and were highlighted for their significant contribution (*p* value <0.05 uncorrected) towards a certain skin colour category (see Table 2). An illustration of the performance of the chosen 5-category and 3-category model and AUC estimates on the total 100% set can be seen in Fig. 2.

**Table 2:**
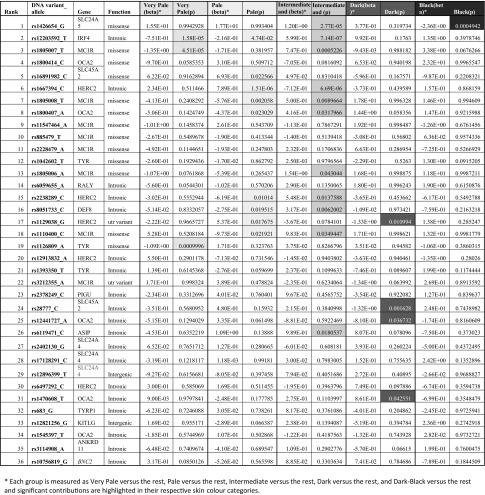
Contribution of each of the 36 selected SNP predictors of skin colour towards binomial prediction categories in terms of the beta coefficients and its statistical significance, within the 5-category skin colour prediction model

However, as the use of 100% of the samples is likely to overestimate the model’s prediction accuracy, the total data set was split 1000 times into 80% training sets (*n* = 1138) and 20% testing sets (*n* = 285) and reassessed by performing cross validations (CV). The resulting average AUC values with standard deviation achieved for the different skin colour categories represent the true model performance assessment, and were 0.74 ± 0.05 for Very Pale, 0.72 ± 0.03 for Pale, 0.73 ± 0.03 for Intermediate, 0.87 ± 0.1 for Dark, and 0.97 ± 0.03 for Dark-Black. For the 3-category model, the achieved average AUC values with standard deviation were 0.97 ± 0.02 for Light, 0.83 ± 0.11 for Dark, and 0.96 ± 0.03 for Dark-Black.

Although the lower values in the Very Pale, Pale, and Intermediate categories reflect a dispersal of the Light category into three separate sub-categories, the prediction model factors in this variation to differentiate individuals that display obvious skin colour differences, i.e., very pale skin versus more ‘olive’ tones. Each category provides additional information on the tanning ability of that predicted individual, which is particularly relevant for predicting the variation seen within Europe, especially when comparing northern to southern Europeans. For instance, although they yield lower independent AUC values, taken collectively together in terms of their probability, they provide additional information overall on whether the individual will remain light or pale skinned all year round (as is the case with Pale to Very Pale high probability estimates) or could potentially darken with tanning (representative of high intermediate category probability estimations). In these cases, one must also consider the time of the year (i.e., summer/winter) on whether an individual could potentially appear darker due to sun exposure or remain the same due to lack of sun exposure.

The models established in this study illustrate the reasonably high degree of categorical skin colour prediction accuracy achieved with this set of 36 SNPs from 16 genes. Not only are the models on both a 3 and 5-category level capable of separating light versus dark skin colours between continental groups, but, moreover, the 5-category model also has the ability to separate the subtle variation observed within continental groups, as observed in the Light category expanding to Very Pale, Pale, and Intermediate category predictions.

### Comparison with previously reported set of skin colour DNA predictors

To directly compare the skin colour prediction result of our newly established model based on a set of 36 SNPs with that of the 10 SNP set skin classifier previously reported by Maroñas et al. (2014), we genotyped a total of 42 SNPs (4 SNPs overlap between the 36 and the 10 SNPs) in an independent set of 194 samples from individuals living in the US (see online resource information) not previously used in selecting the set of SNP predictors nor for the previous model building and testing. For this analysis, we collected skin colour data from these 194 individuals using a handheld Konica Minolta spectrophotometer CM700d and assigned three skin colour categories White, Intermediate, and Black using CIE L*ab values in the same way as previously described by Maroñas et al. (2014). Of the 194 individuals, 131 (68%) individuals were assigned White, 43 (22%) samples were assigned Intermediate, and 20 (10%) samples were assigned Black. When using the 10 SNP set skin classifier from Maroñas et al. (2014), the achieved AUC values were 0.79 for White, 0.63 for Intermediate, and 0.64 for Black.

However, when using our newly proposed model, an improvement in AUC was observed for White (Light) from 0.79 to 0.82, comparable at the Intermediate (Dark) level, from 0.63 to 0.62, and a large increase for Black (Dark-Black) from 0.64 to 0.92 (see Table 3). It should be mentioned, however, that the improved yet low values for the 36-SNP do not reflect the true performance of the model, as the 36 SNP predictors highlighted in the present study were identified using Fitzpatrick scale phenotypes, not using the phenotype scale previously applied by Maroñas et al. (2014) and what is used in this comparative analysis. If, however, the 194 individuals were assessed according to Fitzpatrick-based skin colour categories, Light, Dark, and Dark-Black accuracy levels increase further to 0.92, 0.74, and 0.94 AUC, respectively (see Table 3). Finally, it is believed that the addition of skin colour specific prediction markers is not solely responsible for the large increase in the Black category prediction between models. The increase could also be inflated by the low numbers of Black individuals used for training of the Bayesian classifier model (*n* = 22), especially considering their use of prior odds where allele combinations of individuals from a more global ‘Black’ category would not be wholly represented. In any case, these results indicate that our newly proposed model based on a set of 36 skin colour predicting SNPs outperformed the previously proposed model based on a set of 10 SNPs published by Maroñas et al. (2014) regarding prediction accuracy of skin colour from DNA.

**Table 3:** Model performance comparison of the 10-SNP set Bayes Classifier by Maroñas et al. (2014) and the 36-SNP set prediction model from the present study using the independent “model comparison set” of 194 individuals from 17 populations not previously used for marker discovery by applying the same phenotyping method previously employed by Maroñas et al. (2014) to allow direct comparison of the two prediction approaches

	AUC	Sensitivity	Specificity	PPV	NPV
Bayes classifier 10-SNP model Maroñas et al. (2014)					
White	0.79	0.97	0.62	0.84	0.91
Int	0.63	0.37	0.88	0.47	0.83
Black	0.64	0.30	0.98	0.67	0.92
36-SNP set model current study					
White	0.82	0.99	0.65	0.86	0.98
Int	0.62	0.26	0.98	0.79	0.82
Black	0.92	0.90	0.94	0.64	0.99
36-SNP set model current study—Fitzpatrick scale*					
Light	0.92	0.99	0.85	0.95	0.98
Dark	0.74	0.50	0.99	0.86	0.93
Dark-Black	0.94	0.92	0.96	0.79	0.99

* The 36-SNP set model performance assessment using Fitzpatrick scale phenotypes as the observed phenotype

**Fig. 3:**
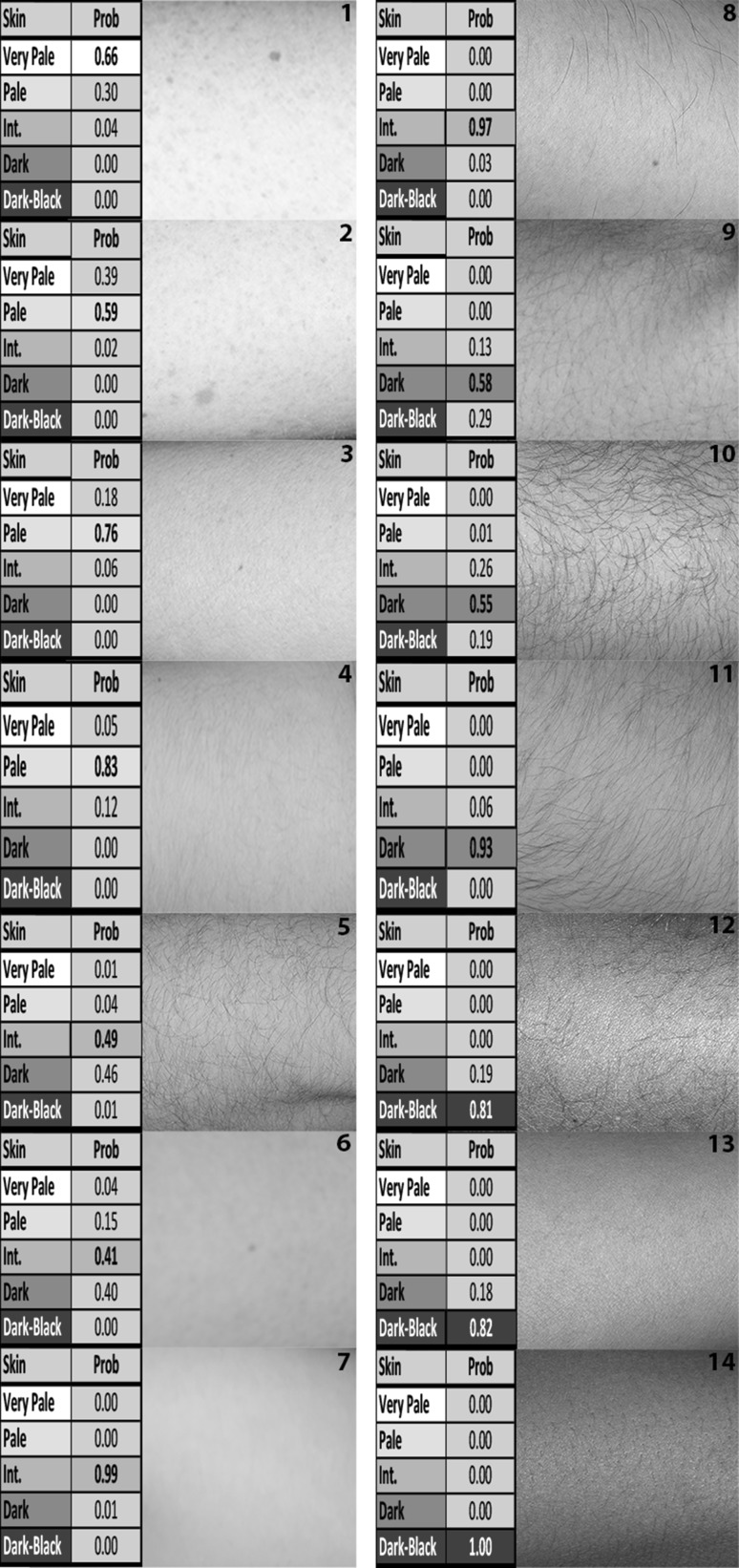
Proof-of-principle illustration of the power of the developed model for predicting skin colour on a global scale, regardless of bio-geographic ancestry. Probability outputs from the 5-category skin colour prediction model based on genotypes of the 36 SNP set are shown together with a skin image of the respective DNA donor. Fourteen individuals were chosen from the ‘model comparison set’ based on their parental country of birth, both in and outside the US, representing globally distributed individuals. The order of the images is 1–14 with the following parental birth countries recorded 1-US, 2-US, 3-US, 4-US, 5-Syria, 6-Columbia, 7-China, 8-Vietnam, 9-El Salvador, 10-India, 11-Mexico, 12-Nigeria, 13-Vietnam, 14-Nigeria

Finally, to provide a proof-of-principle on the final markers chosen for a global skin colour prediction model and the data set used to train the model, 14 individuals were selected from the ‘model comparison set’ (not previously involved in modelling), and the 5-category scale skin colour probabilities are shown together with a skin image (Fig. 3). The individuals were chosen to represent different countries around the world where their birth parents were born in and outside the US. It should be noted that considering the highest two categorical probabilities (and not only the highest one) seem to best reflect the colour palette of that particular individual. These preliminary data indicate that the DNA markers and the prediction model we have developed in this study may achieve DNA-based global skin colour prediction regardless of bio-geographic ancestry, which, however, requires further investigation in additional individuals from around the world. In addition, as with all pigmentation traits, a move to a more continuous skin colour prediction would inevitably improve accuracy overall. However, additional global skin colour markers must be unearthed first via large-scale GWAS’s.

The current prediction model is based on multinomial logistic regression, which included a set of carefully selected SNPs. Prediction modeling using alternative approaches, such as the derivation of polygenic scores based on weighted allele sums using an extended list of trait-associated SNPs, may or may not provide higher prediction accuracies as it depends on the number of added SNPs that actually have low to no association/predictive effects. Moreover, the low quality and quantity of DNA typically obtained in applications using DNA-based prediction of visible traits, such as extracts from teeth or bones in anthropological applications and crime scene traces in forensic applications, typically do not allow the analyses of large numbers of SNPs. Therefore, the use of microarray technology is not optimal, and thus, a targeted approach, such as the genotyping of a limited set of DNA markers, recommended here for skin colour prediction, is currently the preferred method of choice.

## Conclusions

Overall, we demonstrate that global skin colour, between and within continental groups, can be accurately predicted from DNA using a set of 36 carefully selected SNPs from 16 genes. The DNA markers and the model introduced here deliver prediction accuracies already high enough for practical applications, although for the three different light skin colour categories, they may be further improved with additional (but currently unknown) SNP predictors once identified via future GWAS’s. We envision that if combined with the previously established eye and hair colour predicting SNPs, such as those from the IrisPlex and HIrisPlex systems, all three human pigmentation traits can be reliably predicted from DNA in future forensic and anthropological applications.

## Electronic supplementary material

Below is the link to the electronic supplementary material.

Supplementary material 1 (PDF 101 kb)

Supplementary material 2 (PDF 5166 kb)
