# Supplementary material for: Global skin colour prediction from DNA
Source: Hum Genet. 2017 May 12;136(7):847–63. doi: 10.1007/s00439-017-1808-5 (PMC5487854; doi:10.1007/s00439-017-1808-5)
Supplement: Supplementary file 1 — Supplementary material 1 (PDF 101 kb) [file 439_2017_1808_MOESM1_ESM.pdf]

Global skin colour prediction from DNA

Susan Walsh[1,*], Lakshmi Chaitanya[2], Krystal Breslin[1], Charanya Muralidharan[1], Agnieszka Bronikowska[3], Ewelina Pospiech[4,5], Julia Koller[2], Leda Kovatsi[6], Andreas Wollstein[7], Wojciech Branicki[5,8], Fan Liu[2,9,10] and Manfred Kayser[2,*]

[1] Department of Biology, Indiana University Purdue University Indianapolis (IUPUI), Indiana, USA

[2] Department of Genetic Identification, Erasmus MC University Medical Centre Rotterdam, The Netherlands

[3] Department of Dermatology, Collegium Medicum of the Jagiellonian University, Kraków, Poland

[4] Institute of Zoology, Faculty of Biology and Earth Sciences, Jagiellonian University, Kraków, Poland

[5] Malopolska Centre of Biotechnology, Jagiellonian University, Kraków, Poland

[6] Laboratory of Forensic Medicine & Toxicology, School of Medicine, Aristotle University of Thessaloniki, Greece.

[7] Section of Evolutionary Biology, Department of Biology II, University of Munich LMU, Planegg-Martinsried, Germany.

[8] Central Forensic Laboratory of the Police, Warsaw, Poland.

[9] Key Laboratory of Genomic and Precision Medicine, Beijing Institute of Genomics, Chinese Academy of Sciences, Beijing, China

[10] University of Chinese Academy of Sciences, Beijing, China

* Corresponding authors

SW: phone +1-317-274-0593, e-mail walshsus@iupui.edu

or

MK: phone +31-10-7038073, e-mail m.kayser@erasmusmc.nl

Supplementary Material - Online Resource Information 1

Samples and Skin Colour Phenotyping

Country	Very Pale	Pale	Intermediate	Dark	Dark-Black
**Ireland (n=347)**	**65**	**208**	**70**	**3**	**1**
**Poland (n=1159)**	**34**	**543**	**582**	**0**	**0**
**Greece (n=119)**	**4**	**19**	**80**	**16**	**0**
**Other Europeans/Russia (n=5)**	**1**	**0**	**4**	**0**	**0**
**USA* & Canada (n=262)**	**26**	**114**	**91**	**8**	**23**
**South America/Haiti (n=20)**	**0**	**2**	**7**	**10**	**1**
**Middle East*/India (n=22)**	**0**	**0**	**3**	**16**	**4**
**China*/Korea/Vietnam (n=15)**	**0**	**0**	**9**	**6**	**0**
**Africa/Papa New Guinea (n=75)**	**0**	**0**	**0**	**0**	**75**

*** Individuals with admixture indicated in questionnaire data**

Specific Countries in more detail (n=31):
Ireland, Greece, Senegal, Nigeria, Kenya, Papa New Guinea, USA, Mexico, Argentina, Columbia, India, Bangladesh, Cuba, Poland, Palestine, Canada, China, Honduras, Germany, Philippines, Russia, Sudan, Japan, Saudi Arabia, Pakistan, El Salvador, Spain, Haiti, South Korea, Vietnam
