# Supplementary material for: Global skin colour prediction from DNA
Source: Hum Genet. 2017 May 12;136(7):847–63. doi: 10.1007/s00439-017-1808-5 (PMC5487854; doi:10.1007/s00439-017-1808-5)
Supplement: Supplementary file 2 — Supplementary material 2 (PDF 5166 kb) [file 439_2017_1808_MOESM2_ESM.pdf]

Global skin colour prediction from DNA

Susan Walsh[1,*], Lakshmi Chaitanya[2], Krystal Breslin[1], Charanya Muralidharan[1], Agnieszka Bronikowska[3], Ewelina Pospiech[4,5], Julia Koller[2], Leda Kovatsi[6], Andreas Wollstein[7], Wojciech Branicki[5,8], Fan Liu[2,9,10] and Manfred Kayser[2,*]

[1] Department of Biology, Indiana University Purdue University Indianapolis (IUPUI), Indiana, USA

[2] Department of Genetic Identification, Erasmus MC University Medical Centre Rotterdam, The Netherlands

[3] Department of Dermatology, Collegium Medicum of the Jagiellonian University, Kraków, Poland

[4] Institute of Zoology, Faculty of Biology and Earth Sciences, Jagiellonian University, Kraków, Poland

[5] Malopolska Centre of Biotechnology, Jagiellonian University, Kraków, Poland

[6] Laboratory of Forensic Medicine & Toxicology, School of Medicine, Aristotle University of Thessaloniki, Greece.

[7] Section of Evolutionary Biology, Department of Biology II, University of Munich LMU, Planegg-Martinsried, Germany.

[8] Central Forensic Laboratory of the Police, Warsaw, Poland.

[9] Key Laboratory of Genomic and Precision Medicine, Beijing Institute of Genomics, Chinese Academy of Sciences, Beijing, China

[10] University of Chinese Academy of Sciences, Beijing, China

* Corresponding authors

SW: phone +1-317-274-0593, e-mail walshsus@iupui.edu

or

MK: phone +31-10-7038073, e-mail m.kayser@erasmusmc.nl

Supplementary Material - Online Resource Information 2

The Fitzpatrick Scale

The scale was established in 1975 by a dermatologist – Thomas B. Fitzpatrick in an effort to better classify people with white skin for the treatment of particular ailments (psoriasis).

Classifications are based on exposure to UV light. The following table and figure illustrate these categories.

Skin Colour (Unexposed Skin)	Skin Type	Sunburn	Tan
White	I	Yes	No
	II	Yes	Minimal
	III	Yes	Yes
	IV	No	Yes
Brown	V	No	Yes
Black	VI	No	Yes

Taken from Table 2 – Sun Reactive Skin Types in "The validity and practicality of sun-reactive skin types I through VI" (Fitzpatrick 1988)

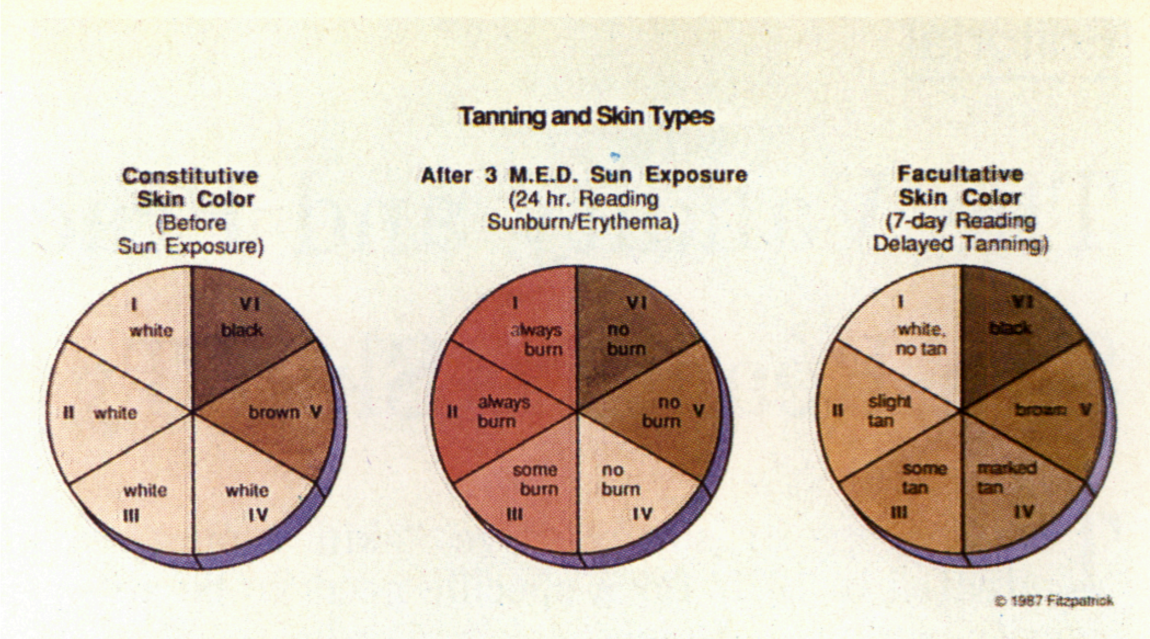

Figure taken from "The validity and practicality of sun-reactive skin types I through VI" (Fitzpatrick 1988) titled 'Tanning and skin types'.

For the prediction categories used in this paper, Fitzpatrick categories were followed for categories I, II, V and VI and renamed Very Pale, Pale, Dark and Dark-Black.
For the Fitzpatrick categories III and IV, these were merged due to their highly similar phenotype to propose an intermediate category for prediction.

The following figure illustrates the prediction categories as proposed by this paper and their comparison to the Fitzpatrick scale assessments.

**Fitzpatrick Scale**	**5 Category Prediction Scale**	**3 Category Prediction Scale**	**Simple visual representation of each Category**
I	Very Pale	Light	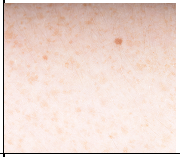
II	Pale	Light	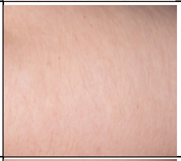
III/IV	Intermediate	Light	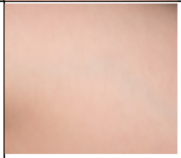
V	Dark	Dark	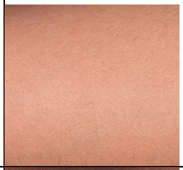
VI	Dark-Black	Dark-Black	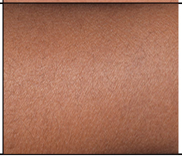
